# Molecular Anatomy of the EML4-ALK Fusion Protein for the Development of Novel Anticancer Drugs

**DOI:** 10.3390/ijms24065821

**Published:** 2023-03-18

**Authors:** So Yeong Cheon, Sunghark Kwon

**Affiliations:** 1 Department of Biotechnology, Konkuk University, Chungju 27478, Republic of Korea; 2 Research Institute for Biomedical & Health Science, Konkuk University, Chungju 27478, Republic of Korea

**Keywords:** echinoderm microtubule-associated protein-like 4, anaplastic lymphoma kinase, EML4-ALK fusion, non-small-cell lung cancer, kinase inhibitor

## Abstract

The *EML4* (echinoderm microtubule-associated protein-like 4)-*ALK* (anaplastic lymphoma kinase) fusion gene in non-small-cell lung cancer (NSCLC) was first identified in 2007. As the EML4-ALK fusion protein promotes carcinogenesis in lung cells, much attention has been paid to it, leading to the development of therapies for patients with NSCLC. These therapies include ALK tyrosine kinase inhibitors and heat shock protein 90 inhibitors. However, detailed information on the entire structure and function of the EML4-ALK protein remains deficient, and there are many obstacles to overcome in the development of novel anticancer agents. In this review, we describe the respective partial structures of EML4 and ALK that are known to date. In addition to their structures, noteworthy structural features and launched inhibitors of the EML4-ALK protein are summarized. Furthermore, based on the structural features and inhibitor-binding modes, we discuss strategies for the development of novel inhibitors targeting the EML4-ALK protein.

## 1. Introduction

According to GLOBOCAN in 2020, lung cancer accounts for approximately 14.3% and 8.4% of male and female cancers diagnosed worldwide, respectively [1]. In general, lung cancer is histologically classified into two groups: small-cell lung cancer (SCLC) and non-small-cell lung cancer (NSCLC). NSCLC is further categorized into three groups: squamous-cell carcinoma, adenocarcinoma, and large-cell carcinoma [2]. NSCLC accounts for more than 80% of all lung cancer cases, most of which are adenocarcinomas [3]. NSCLC is caused by mutations in the epidermal growth factor receptor (EGFR)-encoding gene and abnormal gene fusion, such as that of *EML4* encoding echinoderm microtubule-associated protein-like (EML)4 and *ALK* encoding anaplastic lymphoma kinase (ALK) [4,5,6,7,8].

The oncogenic fusion of *EML4* and *ALK* in NSCLC was first identified in 2007 [8]. As the first EML protein family, echinoderm microtubule-associated protein (EMAP) was identified in sea urchins [9]. EMAP is associated with the regulation of microtubule assembly during mitosis [9]. In the human genome, a total of six *EML* genes (*EML1-6*) encoding EML proteins were identified [10]. ALK is a tyrosine kinase receptor belonging to the insulin receptor superfamily [11,12,13,14,15,16,17]. ALK was first identified as a component of the fusion protein with nucleophosmin in anaplastic large-cell lymphoma [12]. Although the role of ALK is not fully understood, ALK is known to regulate the development of the central and peripheral nervous systems [18]. The EML4-ALK fusion protein plays an abnormal role in the cellular signaling pathway, resulting in excessive cell growth and proliferation [19,20,21,22]. As the EML4-ALK protein has a kinase domain as a component of the protein, much attention has been paid to kinase inhibitors as therapeutic agents for NSCLC [23]. Hence, lung cancer patients with the EML4-ALK protein have been treated with ALK inhibitors [20,24,25,26,27]. In addition, not less than fifteen different variants of EML4-ALK have been identified [8,21,28,29,30,31]. These variants include V1, V2, V3a, V3b, V4, V4′, V5a, V5b, V5′, V6, V7, V8a, V8b, etc. Thus, various genetic combinations for the *EML4-ALK* gene fusion are possible. This implies that patients with EML4-ALK may be clinically treated according to their genetic variant.

Several structures of ALK in complex with their inhibitors have been elucidated using X-ray crystallography [32,33,34,35,36,37,38,39]. These include tyrosine kinase inhibitors such as crizotinib [32,33,34], ceritinib [35], alectinib [36], brigatinib [37], lorlatinib [38], and entrectinib [39]. On the other hand, in the case of EML4, only a few domain structures have been determined [40,41] because the entire structure seems difficult to determine; this is probably owing to the intermediate disordered region. Although the entire EML4-ALK protein structure has not yet been determined, the hitherto known structures enable us to reconstruct a possible entire structure of the EML4-ALK protein.

To date, inhibitors of the EML4-ALK protein have mainly targeted the ALK protein; hence, commercially available inhibitors of the EML4-ALK protein are tyrosine kinase inhibitors [20,24,25,26,27]. However, these tyrosine kinase inhibitors for ALK cause a severe problem of resistance to ALK inhibitors [42,43,44,45,46]. Therefore, it is necessary to develop novel tyrosine kinase inhibitors to circumvent resistance to existing ALK inhibitors. Moreover, we need to find a new strategy for blocking ALK function by targeting its novel drug-binding sites, except for the ATP-binding site.

Here, we describe the known structures of ALK and its complex with inhibitors to obtain structural insights into the inhibitory mechanism. Based on the structural information on the EML4-ALK protein, along with biophysical analysis, we summarize and assess commercial tyrosine kinase inhibitors for ALK. In addition, potent inhibitor-binding sites of the EML4-ALK protein are addressed to craft strategies for the development of novel inhibitors. This review advances our understanding of the molecular biology of the EML4-ALK protein and can ultimately lead to the development of novel anticancer agents.

## 2. Genetic Composition of the *EML4*-*ALK* Fusion Gene

**Figure 1:**
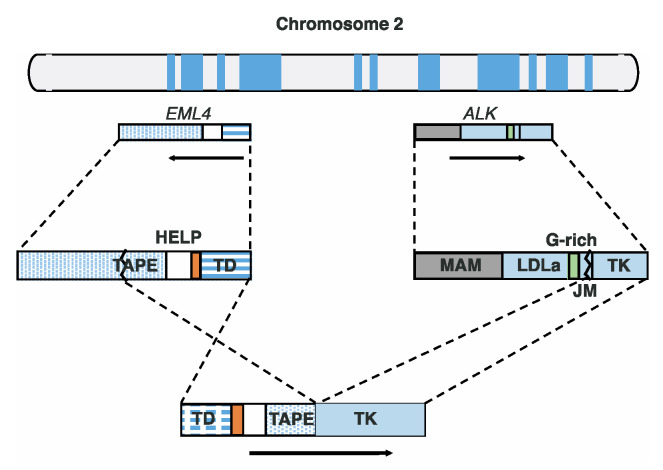
*EML4*-*ALK* gene fusion. The two genes are positioned on the short arm of chromosome 2. The arrow directions indicate the directions of gene expression.

The *EML4* and *ALK* genes are located in the short arm of human autosomal chromosome 2 and in directions opposite to each other (Figure 1) [8]. Upon gene fusion, the *EML4*-*ALK* gene is formed through paracentric inversion [8]. While the *ALK* gene maintains the transcriptional direction, the *EML4* gene undergoes an inversion for gene fusion (Figure 1). To date, at least fifteen EML4-ALK variants have been identified, including multiple isoforms such as splice variants [8,21,28,29,30,31]. In addition, *EML4* and *ALK* genes show striking breakpoint features [8,21,28,29,30,31]. All variants include the tyrosine kinase (TK) domain in ALK because the *ALK* gene is cleaved immediately before the TK domain upon gene fusion. However, breakpoints on the *EML4* gene are variable, and eight breakpoints on the *EML4* gene have been identified thus far [8,21,28,29,30,31]. As a result, these genetic variants lead to structural diversity in the EML4-ALK fusion protein.

The EML4 protein consists of four parts: the tandem atypical propeller domain (TAPE); the hydrophobic motif in EML protein (HELP); the basic region; and the trimerization domain (TD) [10]. The TAPE domain has various breakpoints for EML4-ALK fusion [8,21,28,29,30,31]. The TD domain plays a vital role in ALK autophosphorylation and activation [40]. The ALK protein comprises six major parts: (1) the Meprin, A5 protein, and protein tyrosine phosphatase Mu domain (MAM); (2) low-density lipoprotein receptor class A (LDLa); (3) the glycine-rich region (G-rich); (4) the transmembrane helix (TM); (5) the juxtamembrane domain (JM); and (6) the tyrosine kinase domain (TK) [12]. The JM domain, located immediately before the TK domain, has only one breakpoint. Accordingly, all EML4-ALK variants have the TK domain that originates from ALK. As a result, the EML4-ALK protein simultaneously shows diversity and uniformity in terms of structural composition.

## 3. Structural Analysis of EML4-ALK

### 3.1. Overall Architecture of EML

There are six kinds of *EML* genes (*EML*1-6) in the human genome [10,47,48,49,50]. The EML1-4 proteins share a common structural feature. Namely, they have a TD domain at the N terminus and HELP and TAPE domains at the C terminus. Iterative WD (Trp-Asp) regions are observed in the TAPE domain. The basic region connects the TD domain to HELP and TAPE domains. In contrast, the EML5-6 proteins are devoid of the TD domain and have three repeated TAPE domains [51]. Several partial structures of human EML proteins have been determined [40,41]. The crystal structure of the TAPE domain of EML1 was determined (PDB ID: 4CI8) [40]. The structures of the TD domains of EML2 (PDB ID: 4CGB) [41] and EML4 (PDB ID: 4CGC) [41] have also been elucidated. Although the entire EML structure has not yet been determined, these partial structures provide valuable information, enabling us to assume the entire working mechanism of EML.

**Figure 2:**
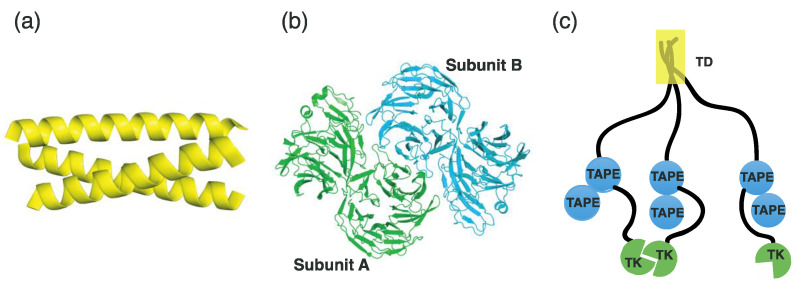
EML4 structure. The TD (**a**) and TAPE (**b**) domains of EML4 are represented as a cartoon. (**c**) The putative entire structure of trimeric EML4-ALK. The yellow box indicates the three TD domains.

Here, we briefly describe the TD domain structure of EML4 and the TAPE domain structure of EML1, which is structurally homologous to EML4. Three TD domains form a bundle of α-helices, creating a coiled-coil architecture (Figure 2a). The TAPE domain comprises a pair of continuous β-propellers (Figure 2b). The respective WD repeat regions form an antiparallel β-sheet comprising four β-strands. Seven repeated β-sheet units form the β-propeller architecture. Given that EML4-ALK forms a trimer, owing to the association of three respective TD domains, the TK domains from ALK are prone to dimerization, leading to autophosphorylation for activation (Figure 2c).

### 3.2. TK Domain Structure of ALK

Human ALK consists of 1620 amino acids, of which the C-terminal region (amino acids 1096–1394) corresponds to the TK domain. In contrast to EML4, ALK has only one breakpoint immediately prior to the TK domain [8]. Thus, all the EML4-ALK variants share the TK domain at the C-terminal region. Therefore, considering that most EML4-ALK anticancer agents target the TK domain, the structure of the TK domain should be analyzed at the molecular level.

To date, several crystal structures of TK, including its native and inhibitor complex forms, have been determined. All the native structures have point mutations, including L1196M (PDB ID: 2YHV [33]), F1174L (PDB ID: 2YJR), C1156Y (PDB ID: 2YJS), G1269A (PDB ID: 4ANL [33]), F1174L (PDB ID: 4FNW [52]), and R1275Q (PDB ID: 4FNX [52]). A TK structure in complex with ADP, one of the products (PDB ID: 3LCT), was also elucidated [53]. This structure provides valuable information on its product-binding mode, which enables us to surmise its substrate-binding mode. In addition, structures in complex with inhibitors have also been determined. The inhibitors in these complex structures include crizotinib (PDB ID: 2XP2 [32], 2YFX [33], 4ANQ [33], 4ANS, 5AAA [34], 5AAB [34], and 5AAC [34]); ceritinib (PDB ID: 4MKC [35] and 4UXL [54]); alectinib (PDB ID: 3AOX [36]); brigatinib (PDB ID: 6MX8 [37]); lorlatinib (PDB ID: 4CLI [38], 4CLJ [38], 5AAU [34], 5AA8 [34], and 5AA9 [34]); and entrectinib (PDB ID: 5FTO [39]). These structures show how the respective inhibitors bind to the ATP pocket, leading to the development of novel ALK inhibitors. In this section, we describe the structure of the TK domain containing ADP (PDB ID: 3LCT [53]).

**Figure 3:**
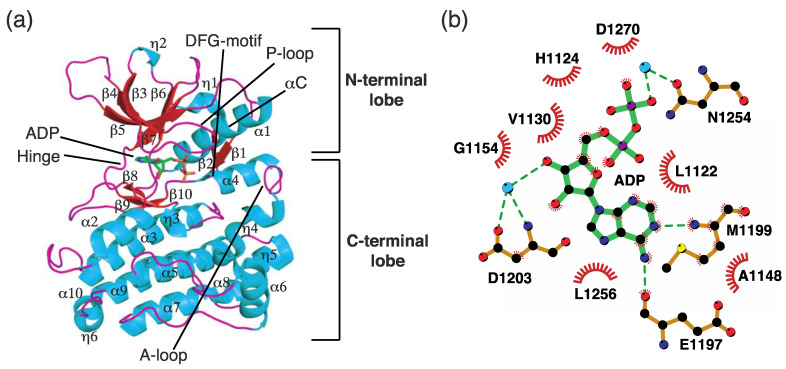
The TK domain structure of ALK. (**a**) Crystal structure of the TK domain. (**b**) Schematic diagram of ADP showing interactions with adjacent residues. The cyan circles and green dashed lines indicate water molecules and hydrogen bonds, respectively.

The TK domain is composed of two lobes, an N-terminal lobe and a C-terminal lobe (Figure 3a). The N-terminal lobe contains an α-helix (α1), two 3_10_-helices (η1–2), seven β-strands (β1–7), part of which form an antiparallel β-sheet (β1–5), and several loops, whereas the C-terminal lobe comprises nine α-helices (α2–10), four 3_10_-helices (η3–6), three β-strands (β8–10), and several loops. The N-terminal lobe is connected to the C-terminal lobe by a hinge region. The active site is formed between the N- and C-terminal lobes, and the hinge region is involved in the formation of the active site (Figure 3a).

The ATP-binding pocket, which is a part of the active site, is composed of several important components. A loop connecting the β3 and β4 strands is called a P-loop or G-loop (Gly-rich loop) (Figure 3a). This loop exhibits considerable flexibility and adopts an appropriate conformation in response to substrate binding. The α1 helix is located next to the β-sheet and is called the C-helix (αC) (Figure 3a). The C-helix can adopt two different conformations by moving inwards (αC-in) or outwards (αC-out). In the αC-in state, Gly1167 in the C-helix forms a salt bridge with Lys1150 in the β3 strand, thereby contributing to the formation of the active site that is suitable for catalysis. The DFG motif is positioned between β10 and α4 (Figure 3a). This region is also conserved among the tyrosine kinases. The DFG motif plays a vital role in catalysis by adopting two different conformations (DFG-in and DFG-out). In the DFG-in conformation, Asp1270 orients toward the ATP-binding site, thereby coordinating a Mg ion, which ATP usually contains. However, Asp1270 is positioned outwards from the ATP-binding site in the DFG-out conformation, where it does not coordinate the Mg ion. Finally, a relatively long loop called the A-loop (activation loop) corresponds to a region from α4 to η5 (Figure 3a). The A-loop adopts two distinct conformations (active and inactive forms). Although the ADP complex TK structure shows secondary structures such as α4, η4, and η5 in the A-loop, this region exhibits unstructured conformations depending on the molecular environment. In its active form, as shown in Figure 3a, the A-loop is positioned in proximity to the C-helix, thereby facilitating the access of ATP to the ATP-binding site.

### 3.3. Substrate-Binding Mode of the TK Domain for Catalysis

Several crystal structures of the ALK TK domain have been deposited in the PDB, as mentioned Section 3.2. While most of these are structures in complex with ALK inhibitors, only one structure contains ADP (PDB ID: 3LCT [53]). Based on this structural information, we can infer the ATP-binding mode and catalytic mechanism of ALK at the molecular level. Protein kinases catalyze the transfer of the γ-phosphate of ATP to hydroxyl group-containing residues such as serine, threonine, and tyrosine. ALKs target tyrosine as a phosphate acceptor. Therefore, human ALK contains a spatially conserved tyrosine residue. In this section, we describe the ATP-binding mode at the active site for ALK catalysis.

One of the most remarkable hallmarks of ATP binding to the active site is that the adenine ring moiety mainly forms a couple of hydrogen bonds with residues in the hinge region (Figure 3b). Specifically, the N10-bound hydrogen and the N1 nitrogen atoms in the adenine ring form hydrogen bonds with the backbone atoms of Glu1197 and Met1199 in the hinge region. Oxygen atoms in the 5-carbon sugar (O3′) and β-phosphate indirectly interact with Asp1203 and Asn1254, respectively, mediated by adjacent water molecules. In addition, several hydrophobic residues, such as Leu1122, Gly1125, Val1130, Ala1148, and Leu1256, are associated with ADP binding through hydrophobic interactions. Consequently, a local hydrogen bond network, including the hinge region and adjacent water molecules, is assumed to constitute the main mode of ATP binding.

## 4. EML4-ALK Inhibitors

### 4.1. Representative ALK Inhibitors

To date, several ALK inhibitors have been approved and launched in the pharmaceutical market. They are classified into three generations based on their time of development and indications. The human ALK inhibitor profiles are summarized in Table 1.

Crizotinib, the first ALK inhibitor approved by the U.S. Food and Drug Administration (FDA) in 2011, targets ALK and ROS1 to treat metastatic NSCLC and ALK-positive myofibroblastic tumors [55,56]. Crizotinib was developed and launched by Pfizer under the trade name Xalkori in the U.S. Crizotinib is a type I inhibitor, which binds to active forms of ALK. Crizotinib also inhibits the c-Met/Hepatocyte growth factor receptor tyrosine kinase [55,56].

Ceritinib, sold under the brand name Zykadia, targets ALK as a type I inhibitor [57]. Ceritinib was approved in 2014 to treat ALK-positive metastatic NSCLC. It was developed by Novartis. Ceritinib corresponds to a second-generation ALK inhibitor because it inhibits the ALK mutations resistant to crizotinib.

The major targets of alectinib, with the brand name Alecensa, are ALK and RET (a receptor tyrosine kinase) [58]. In 2014, it was first approved to treat ALK-positive NSCLC in Japan. Then, alectinib was granted an accelerated approval by the U.S. FDA in 2015. It was also approved by the European Medicines Agency in 2017.

Brigatinib, with the brand name Alunbrig, targets ALK and the mutated epidermal growth factor receptor [37]. The U.S. FDA granted an accelerated approval in 2017 to treat ALK-positive NSCLC. Brigatinib also inhibits ROS proto-oncogene-1 (ROS1) fusions [37].

Lorlatinib, under the brand name Lorbrena, acts on ALK and ROS1 [59]. Its application is for ALK-positive metastatic NSCLC. Lorlatinib has shown significant clinical effects in patients with the ALK G1202R mutation [59]. Lorlatinib was approved by the U.S. FDA to treat ALK-positive metastatic NSCLC in 2018. It was also approved by the European Commission for the same application.

Entrectinib, sold under the brand name Rozlytrek, selectively inhibits the ALK, ROS1, and tropomyosin receptor kinases [60]. It is used to treat ROS1-positive NSCLC and neutrophic tyrosine receptor kinase fusion-positive cancer. Entrectinib was approved by the U.S. FDA in 2019 and subsequently in Australia in 2020. Entrectinib and lorlatinib are third-generation ALK inhibitors.

### 4.2. Structures of ALK–Inhibitor Complexes

**Figure 4:**
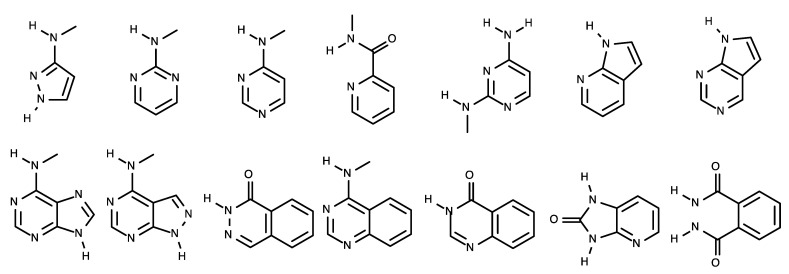
Fourteen common hinge binder scaffolds.

All kinases have an ATP-binding pocket, which an ATP molecule as one of two substrates binds to. This indicates that this ATP-binding mode can be exploited for the development of kinase inhibitors. Indeed, all kinase inhibitors launched in the drug market thus far have been developed based on this binding mode. Specifically, the ATP-binding pocket has a hinge region, where several oxygen and hydrogen atoms forming peptide bonds interact with the adenine moiety of ATP. Accordingly, this adenine component is referred to a hinge binder. In general, a hinge binder consists of hydrogen donors and acceptors and constitutes an essential part of kinase inhibitors. A number of biochemical experiments have shown that this hinge binder in inhibitors plays a crucial role in inhibiting the function of kinases [61]. Typical hinge binder scaffolds for kinase inhibitors are presented in Figure 4.

As discussed in Section 4.1, several ALK inhibitors have been approved and launched in the pharmaceutical market. Structures of ALK–inhibitor complexes have also been reported. These complex structures include crizotinib (PDB ID: 2XP2 [32], 2YFX [33], 4ANQ [33], 4ANS, 5AAA [34], 5AAB [34], and 5AAC [34]); ceritinib (PDB ID: 4MKC [35]); alectinib (PDB ID: 3AOX [36]); brigatinib (PDB ID: 6MX8 [37]); lorlatinib (PDB ID: 4CLI [38], 4CLJ [38], 5AA8 [34], 5AA9 [34], and 5A9U [34]); and entrectinib (PDB ID: 5FTO [39]). This section describes the crystal structures of ALK complexed with its respective inhibitors.

#### 4.2.1. Binding Mode of the ALK–Crizotinib Complex

**Figure 5:**
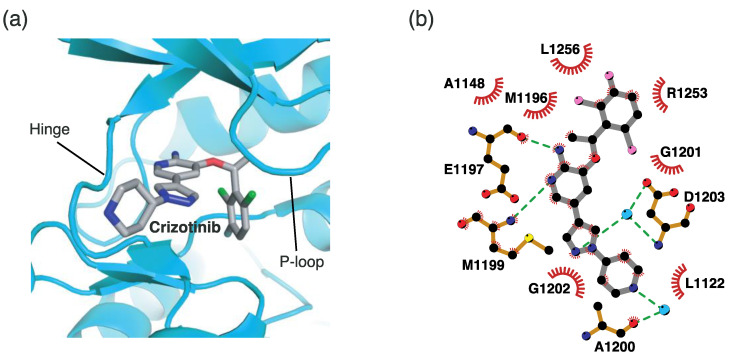
Crizotinib-binding mode. (**a**) Crystal structure of the TK domain (cyan) in complex with crizotinib. Carbon, nitrogen, oxygen, chlorine, and fluorine atoms are colored gray, blue, red, green, and cyan, respectively. (**b**) Schematic diagram of crizotinib showing interactions with adjacent residues. The circle and line codes are the same as in Figure 3b.

Crizotinib is a first-generation ALK inhibitor. The 2-aminopyridine moiety of crizotinib interacts with two residues in the hinge region, as shown in Figure 5. Specifically, the amino group (-NH_2_) hydrogen atom and pyridine N1 nitrogen atom form hydrogen bonds with the backbone oxygen atom of Glu1197 and the backbone N-H hydrogen atom of Met1199, respectively. In addition to this hinge binder interaction, the pyrazole N2 nitrogen atom also forms a hydrogen bond with an adjacent water molecule bound to Asp1203. The piperidine (-NH) hydrogen atom interacts with Ala1200, which is mediated by another water molecule. Additionally, crizotinib hydrophobically interacts with neighboring hydrophobic residues, such as Leu1122, Ala1148, Met1196, Gly1201, Gly1202, Leu1256, and Arg1253.

#### 4.2.2. Binding Mode of the ALK–Ceritinib Complex

**Figure 6:**
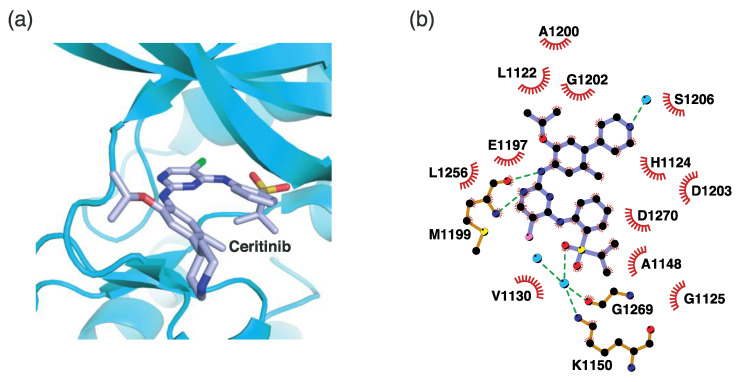
Ceritinib-binding mode. (**a**) Crystal structure of the TK domain (cyan) in complex with ceritinib. Carbon, nitrogen, oxygen, chlorine, and sulfur atoms are colored marine, blue, red, green, and orange, respectively. (**b**) Schematic diagram of ceritinib showing interactions with adjacent residues.

Ceritinib is a second-generation ALK inhibitor. Similarly to the crizotinib-ALK structure, ceritinib interacts with the hinge region (Figure 6). The 2-aminopyrimidine nitrogen and hydrogen atoms form hydrogen bonds with the Met1199 backbone in the hinge region. Specifically, the amino substituent (-NH) hydrogen atom and the pyrimidine nitrogen atom form hydrogen bonds with the backbone oxygen and -NH hydrogen atoms of Met1199, respectively. In addition, one oxygen atom in the sulfone moiety interacts with Lys1150 and Gly1269 via a water molecule. Compared to crizotinib, ceritinib interacts with more adjacent hydrophobic residues. These residues include Leu1122, His1124, Gly1125, Val1130, Ala1148, Glu1197, Ala1200, Gly1202, Asp1203, Ser1206, Leu1256, and Asp1270.

#### 4.2.3. Binding Mode of the ALK–Alectinib Complex

**Figure 7:**
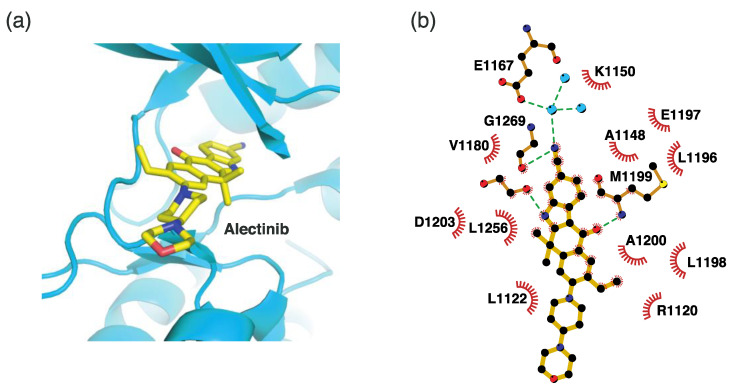
Alectinib-binding mode. (**a**) Crystal structure of the TK domain (cyan) in complex with alectinib. Carbon, nitrogen, and oxygen atoms are colored yellow, blue, and red, respectively. (**b**) Schematic diagram of alectinib showing interactions with adjacent residues.

Alectinib is a second-generation ALK inhibitor. In contrast to crizotinib and ceritinib, alectinib uses a ketone group as the hinge binder, as shown in Figure 7. The oxygen atom of this ketone group forms a hydrogen bond with the Met1199 backbone in the hinge region. Alternatively, the nitrile group interacts with Glu1167 and Gly1269. Alectinib also exploits hydrophobic interactions as the major binding mode. These residues include Arg1120, Leu1122, Ala1148, Lys1150, Val1180, Leu1196, Glu1197, Leu1198, Ala1200, Asp1203, and Leu1256.

#### 4.2.4. Binding Mode of the ALK–Brigatinib Complex

**Figure 8:**
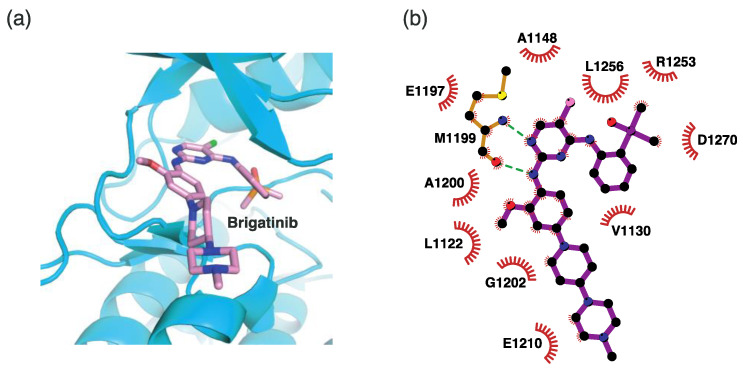
Brigatinib-binding mode. (**a**) Crystal structure of the TK domain (cyan) in complex with brigatinib. Carbon, nitrogen, oxygen, chlorine, and phosphorus atoms are colored pink, blue, red, green, and orange, respectively. (**b**) Schematic diagram of brigatinib showing interactions with adjacent residues.

Brigatinib is a second-generation ALK inhibitor. Similarly to ceritinib, the 2-aminopyrimidine moiety of brigatinib functions as a hinge binder (Figure 8). The nitrogen and hydrogen atoms in 2-aminopyrimidine interact with the Met1199 backbone through hydrogen bonds. Brigatinib also interacts with adjacent hydrophobic residues, such as Leu1122, Val1130, Ala1148, Glu1197, Ala1200, Gly1202, Glu1210, Arg1253, Leu1256, and Asp1270.

#### 4.2.5. Binding Mode of the ALK–Lorlatinib Complex

**Figure 9:**
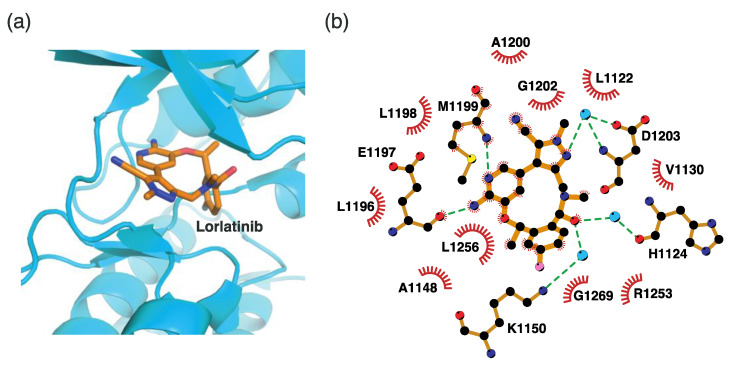
Lorlatinib-binding mode. (**a**) Crystal structure of the TK domain (cyan) in complex with lorlatinib. Carbon, nitrogen, oxygen, and fluorine atoms are colored orange, blue, red, and cyan, respectively. (**b**) Schematic diagram of lorlatinib showing interactions with adjacent residues.

Lorlatinib is a third-generation ALK inhibitor. It is the only organic heterotetracyclic compound of ALK inhibitors. This unique chemical structure shows a different binding mode from that of other ALK inhibitors. In the lorlatinib structure, the 2-aminopyridine moiety functions as a hinge binder by interacting with Glu1197 and Met1199 in the hinge region (Figure 9). In addition, the amide oxygen atom interacts with His1124 and Lys1150 via two water molecules. The diazole N2 nitrogen atom indirectly interacts with Asp1203 via a water molecule. Consequently, lorlatinib forms more hydrogen bonds for binding to the active site than other ALK inhibitors do. Hydrophobic interactions are also associated with lorlatinib binding. The residues for these interactions correspond to Leu1122, Val1130, Ala1148, Leu1196, Leu1198, Ala1200, Gly1202, Arg1253, Leu1256, and Gly1269.

#### 4.2.6. Binding Mode of the ALK–Entrectinib Complex

**Figure 10:**
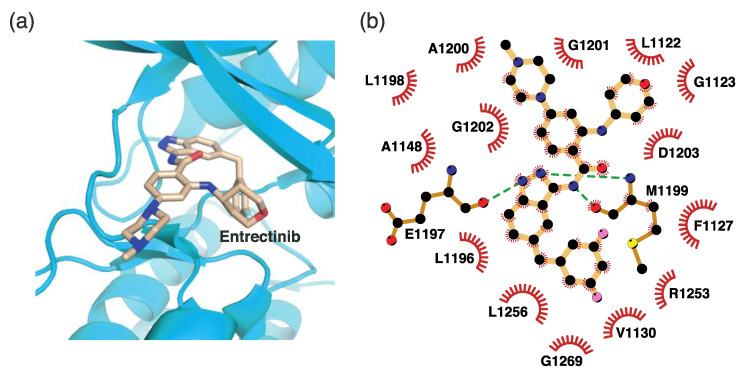
Entrectinib-binding mode. (**a**) Crystal structure of the TK domain (cyan) in complex with entrectinib. Carbon, nitrogen, oxygen, and fluorine atoms are colored wheat, blue, red, and cyan, respectively. (**b**) Schematic diagram of entrectinib showing interactions with adjacent residues.

Entrectinib is a third-generation ALK inhibitor. Unlike lorlatinib, entrectinib uses an indazole moiety as a hinge binder, as shown in Figure 10. The hydrogen atom, which is linked to N1, and the N2 nitrogen atom form hydrogen bonds with Glu1197 and Met1199, respectively. Entrectinib exploits hydrophobic interactions as a major binding mode element. Fourteen neighboring residues are involved in these interactions. These residues include Leu1122, Gly1123, Phe1127, Val1130, Ala1148, Leu1196, Leu1198, Ala1200, Gly1201, Gly1202, Asp1203, Arg1253, Leu1256, and Gly1269. Remarkably, the three-dimensional conformation of entrectinib upon binding to the active site is similar to that of lorlatinib (Figure 9 and Figure 10).

### 4.3. Molecular Mechanism of EML-ALK Inhibitor Resistance

Resistance to inhibitors naturally occurs in kinases over time. Mutations in active site-constructing residues in kinases are frequently observed in cancer cells treated with anticancer chemotherapeutic agents [42,43,44,45,46]. Usually, even a single mutation can reduce the affinity of inhibitors for their binding sites [62]. Specifically, the sites for mutations include the gatekeeper, A-loop, and several other positions [62].

In general, the gatekeeper residue is the most common mutation site among inhibitor-exposed kinases [12,44,63]. Considering that the size and shape of the gatekeeper residue regulate the access of a molecule binding to the hydrophobic back pocket, the affinity of kinase inhibitors to the ATP-binding site can be decreased by mutations in the gatekeeper residue. Several studies have reported gatekeeper mutations in various kinases [45,64,65,66,67,68,69]. As a gatekeeper, the Leu residue is mutated to Met (L1196M) in ALK [45]. At the same position, a Tyr residue is mutated to Ile in BCR-ABL1 (T315I) [64], KIT (T670I) [65], and platelet-derived growth factor receptor (T674I and T681I) [66,67], whereas a Val residue is mutated to Met (V561M) in fibroblast growth factor receptor-1 [68]. In fetal liver kinase-3, the Gly residue is mutated to Arg (G697R) [69].

Considering that a loop has intrinsic flexibility, mutations in the A-loop exhibit greater variability than those in the gatekeeper. Because the inactive conformation of the A-loop is different from the active conformation, novel kinase inhibitors can be designed, based on this inactive conformer. However, mutations in the A-loop maintain the active conformation, thereby reducing the affinity of the inhibitors for the specific binding site in the inactive conformation [70]. Even a single mutation can destroy the inactive conformation of the A-loop [70]. Therefore, mutations in the A-loop constitute another important molecular mechanism for resistance to kinase inhibitors.

The L1196M mutant is a gatekeeper ALK mutant. This mutation stabilizes the active conformation of ALK, resulting in resistance to crizotinib, alectinib, and lorlatinib [45]. This inhibitor resistance results from ALK activation, rather than blocking the access of inhibitors to the ATP-binding site. The L1198F mutant occurs in the hinge region. The replacement of leucine with phenylalanine may hinder inhibitor binding because of the bulkier side chain of phenylalanine. Other mutations are observed in the entrance region of the ATP-binding site. These mutations include G1201R, D1203N, S1206Y, and E1210K [71]. It is unknown how these mutations result in ALK inhibitor resistance. Considering that these residues are located at the entrance of the ATP-binding site, these mutated residues may hinder the access of inhibitors to the binding site. In another scenario, they may expel the A-loop in the inactivated conformation, thereby maintaining the A-loop in the activated conformation. The hydrophobic back pocket is also a mutation site for ALK inhibitor resistance. To date, seven mutations, namely I1171T/N, F1174L/C, V1180L, and G1269A/S, have been identified as hydrophobic back pocket mutations [71]. However, the resistance mechanisms of these mutations remain elusive. These mutated residues might affect adjacent hydrophobic residues, causing local environmental changes that reduce the affinity of ALK inhibitors to the binding pocket. Lastly, the 1151Tins, L1152P/R, and C1156Y/T mutations are located between β5 and the C-helix [71]. These mutations confer resistance to crizotinib and ceritinib.

## 5. Strategies for the Development of Novel EML4-ALK Therapies

Owing to the diversity of EML4 breakpoints for the EML4-ALK fusion, variants with different combinations exist [8,21,28,29,30,31]. Of these, variants 1, 2, 4, 6, 7, and 8 have HELP and TAPE domains in the EML4 region. It is noteworthy that the TAPE domain requires heat shock protein 90 (Hsp90) for structural stabilization [40]. The inhibition of Hsp90 leads to the destabilization of the TAPE domain, eventually inducing the degradation of EML4-ALK through the proteasome. Several studies have reported that EML4-ALK is degraded by Hsp90 inhibition [72,73,74]. Therefore, Hsp90 inhibitors can be used as anticancer agents for EML4-ALK. However, this therapeutic approach is limited to EML4-ALK variants that contain a TAPE domain. Potential Hsp90 inhibitors [75,76,77,78,79,80,81,82] as EML4-ALK therapeutic agents are presented in Table 2.

The A-loop in the TK domain of ALK has been considered an important part for structure-based drug design in the medicinal field to target kinases [62]. The most notable feature is that the A-loop exhibits two different conformations. The A-loop in the activation conformation is located near the C-helix, rendering the ATP-binding site constructed. Numerous kinase inhibitors have been developed based on the structural information on this conformation [32,33,34,35,36,37,38,39]; however, these inhibitors share the same binding site with ATP. This signifies that inhibitors targeting the ATP-binding site cannot specifically bind to other kinases that are not their targets. Such limitations in target specificity raise a fundamental issue regarding the necessity of the development of specific kinase inhibitors.

**Figure 11:**
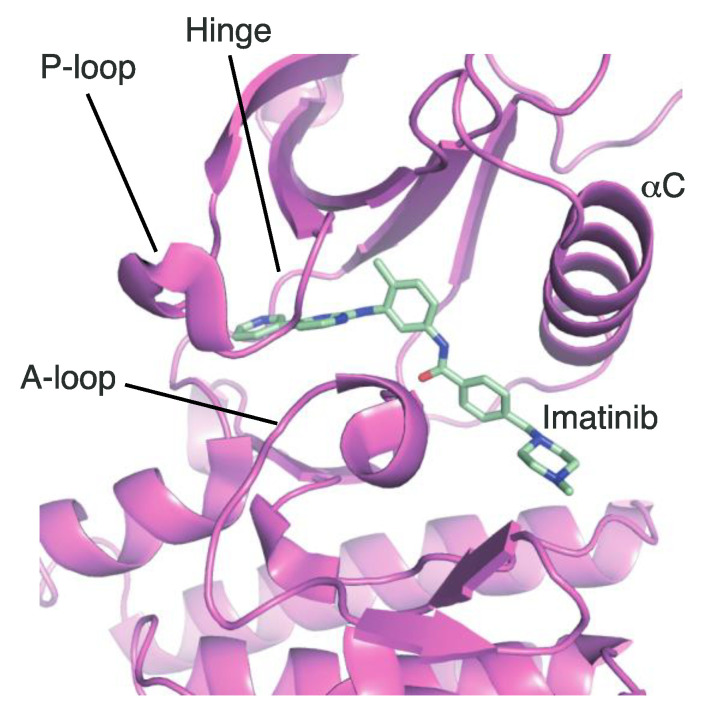
Crystal structure of Abl1 (magenta) in complex with imatinib. Carbon, nitrogen, and oxygen atoms are colored mint, blue, and red, respectively.

In contrast to the activation conformation, the A-loop in the inactivation conformation is located in the proximity of the P-loop. This inactivation conformation creates a spatial cavity different from that in the activation conformation, providing structural information on the binding site of novel ALK inhibitors. However, the inactive conformation of ALK has not been reported. To date, the inactive conformation of Abl1, a non-receptor tyrosine-protein kinase (PDB ID: 1IEP), has been elucidated [83]. If structural information on the inactive conformation of ALK can be obtained, novel ALK inhibitors can be designed and developed, as in the case of imatinib (Figure 11).

**Figure 12:**
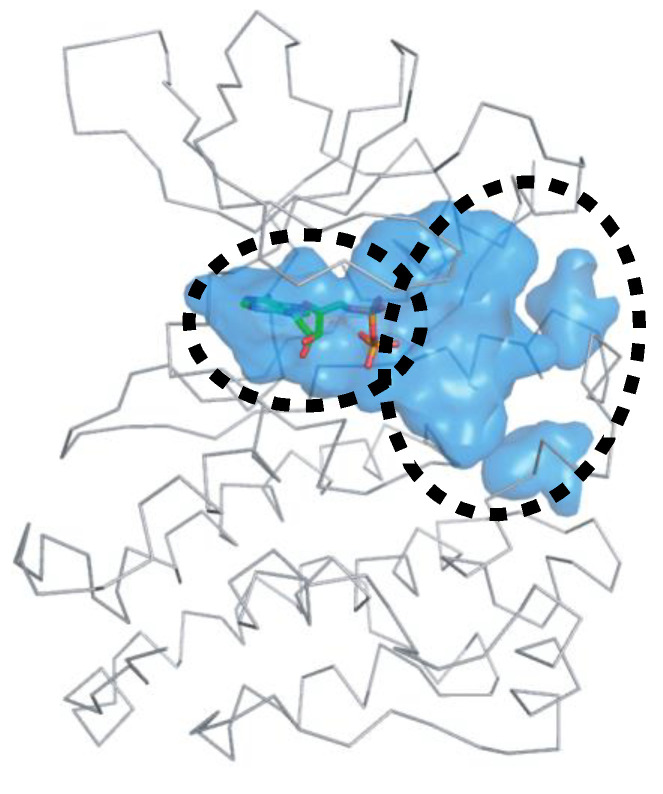
The active site of the ALK TK domain. The protein and ADP structures are represented as ribbon and sticks, respectively. Carbon, nitrogen, oxygen, and phosphorus atoms of ATP are colored green, blue, red, and orange, respectively. The active site is depicted with blue cavities. The left and right dashed ellipses indicate the ATP- and peptide-binding sites, respectively.

Another strategy for the development of novel ALK inhibitors is to exploit the peptide-binding site, including the target Tyr residue. ALK inhibitors have been developed to target the ATP-binding site. However, it is noteworthy that ALK requires two types of substrates for phosphorylation: ATP and Tyr. As shown in Figure 12, the peptide-binding site is located next to the ATP-binding site. The target Y1278 residue for phosphorylation is positioned at the 1278-YRASYY-1283 motif of the A-loop [84]. Accordingly, if the peptide-binding site is occupied by compounds suitable for its volume, trans-phosphorylation is essentially inhibited. Using this strategy, we can design novel inhibitors based on structural information on either the peptide-binding site or the entire active site, including the ATP-binding site.

Finally, blocking the dimerization of the ALK TK domains can be considered. In EM4-ALK, TK dimerization is achieved through the assembly of three TD domains from EML4 (Figure 2a). Accordingly, if the assembly of the TD domains is hindered, dimerization of the TK domains may be inhibited. Indeed, a recent study demonstrated that maintaining the monomeric state of EML4-ALK blocked its dimerization of the ALK TK domain, leading to the suppression of tumor growth both in vitro and in vivo [85]. The authors used synthetic peptides mimicking the TD domain sequences to inhibit the assembly of the TD domains [85]. In future studies, it will be necessary to optimize the size and affinity of synthetic peptides to mimic TD domain sequences. Small compounds that block the trimerization of TD domains can also be considered as alternatives to synthetic peptides.

## 6. Discussion

EML4-ALK is the product of genetic recombination at the chromosomal level. The TK domain of this fusion protein induces continuous autophosphorylation, leading to uncontrolled cell growth and proliferation in NSCLC cells [19,20,21,22]. Because the main function of EML4-ALK in NSCLC cells is kinase activity, the development of anticancer agents for EML4-ALK has focused on inhibition of the catalytic function of the TK domain [32,33,34,35,36,37,38,39]. Specifically, these inhibitors targeted the ATP-binding site in the TK domain. However, similarly to other kinases, EML4-ALK naturally causes mutations in the inhibitor-binding site over time [71]. These mutations often result in resistance to the existing therapies [71]. Therefore, it is necessary to periodically develop novel drugs that target EML4-ALK.

Fortunately, accumulated structural studies on EML4-ALK thus far have provided a structural basis for novel drug targets. As the structure of the TAPE domain of EML1 was determined, the dependence of the TAPE domain on Hsp90 was elucidated [40]. These results indicate that Hsp90 inhibitors can be exploited as anticancer agents for EML4-ALK. The structural information on the A-loop conformations of other kinases homologous to ALK also suggests that the inactivated conformation of the A-loop can form a framework for designing novel inhibitors. Additionally, the A-loop is associated with the formation of a peptide-binding site. Accordingly, understanding the dynamic properties of the A-loop is critical for designing novel inhibitors suitable for specific volumes depending on its different conformations.

Blocking dimerization of the TK domain is an emerging strategy for the development of novel EML4-ALK drugs. Considering that the TD domain plays a pivotal role in inducing dimerization of the TK domain, structural disruption of the trimerization of the TD domain can lead to inhibition of the trans-phosphorylation of the TK domains. Other interaction sites for the dimerization of the TK domain remain elusive, as it is difficult to capture the static state of the dimeric TK domain at the moment of phosphorylation.

Therefore, future studies should focus on understanding the dynamic behavior of EML4-ALK, along with its conformational landscape. Cryogenic electron microscopy (cryo-EM) enables the estimation of different conformations of a specific protein. We may obtain structural information on the disordered region of EML4-ALK using cryo-EM. High-speed atomic force microscopy (HS-AFM) may also be a useful tool for observing the dynamic properties of EML4-ALK. In particular, the different conformations of the A-loop observed through HS-AFM on an adequate timescale can provide valuable information on the structural change in the active site in terms of volumetric analysis. In addition, computational methods such as molecular dynamics simulations can be utilized in cases where experimental feasibility is scarce. A better understanding of the structural biology of EML4-ALK will open a new era of novel EML4-ALK therapies.

## Figures and Tables

**Table 1:** Human ALK inhibitor profiles.

Generation	Generic Name	Trade Name	Chemical Structure	Target	Type	Approval Year	Approval Country	PDB ID ^a^
I	Crizotinib	Xalkori	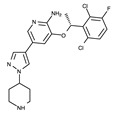	ALK/ROS1	I	2011 (ALK) 2016 (ROS1)	US/EU	2XP2, 2YFX, 4ANQ, 4ANS, 5AAA, 5AAB, 5AAC
II	Ceritinib	Zykadia	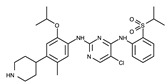	ALK	I	2014	US	4MKC
II	Alectinib	Alecensa	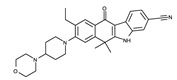	ALK	I	2014/ 2015/ 2017	Japan/ US/ EU	3AOX
II	Brigatinib	Alunbrig	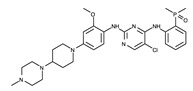	ALK/EGFR	I	2016	US	6MX8
III	Lorlatinib	Lorbrena	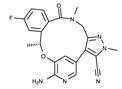	ALK/ROS1	I	2015/2019	US/EU	4CLI, 4CLJ, 5AA8, 5AA9, 5A9U
III	Entrectinib	Rozlytrek	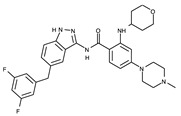	ALK/ROS1/TRK ^b^	I	2019/2020	US/ Australia/EU	5FTO

^a^ Inhibitor complex structure. ^b^ Tropomyosin receptor kinase.

**Table 2:** Representative Hsp90 inhibitor profiles.

Name	Chemical Structure	Potency ^a^	Molecular Weight	Kd	Reference
KW-24783	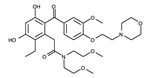	++++ ^b^	574.7	3.8 nM	[75]
Onalespib	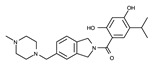	+++ ^c^	409.5	18 nM	[75,76,77]
Ganetespib	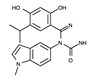	+++	364.4	4 nM	[75,78]
BIIB021	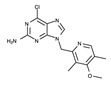	++++	318.8	1.7 nM	[75,79]
Luminespib	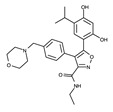	+++	465.5	13 nM	[75,80]
Tanespimycin	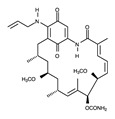	+++	585.7	5 nM	[81]
HSP990	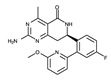	++++	379.4	0.6 nM	[82]

^a^ More plus signs indicate higher potency values, based on Kd values. ^b^ Four plus signs (++++) indicate that Kd value is less than 4 nM. ^c^ Three plus signs (+++) indicate that Kd value is not less than 4 nM.

## Data Availability

Not applicable.
